# Report of Committee on Vital Statistics

**Published:** 1891-01

**Authors:** 


Report of Committee on Vital Statistics, State Board of Health of Penna.

This report suggests an improved method of making out burial certificates. It also contains an appendix giving a list of terms to be used by physicians in describing the cause of death; the object being to bring about greater clearness and uniformity of description in these cases, and thus to facilitate the collection of vital statistics.

